# Transcriptome Profiling Reveals Molecular Changes during Flower Development between Male Sterile and Fertile Chinese Cabbage (*Brassica* *rapa* ssp. *pekinensis*) Lines

**DOI:** 10.3390/life11060525

**Published:** 2021-06-04

**Authors:** Jingfeng Hu, Mei Lan, Xuezhong Xu, Hongli Yang, Liqin Zhang, Fengxian Lv, Huiju Yang, Ding Yang, Chongjuan Li, Jiangming He

**Affiliations:** 1 Institute of Horticultural Crops, Yunnan Academy of Agricultural Sciences, Yunnan Branch of the National Vegetable Improvement Center, Kunming 650205, China; maxgboy@163.com (J.H.); personal_miss@163.com (M.L.); xzhxu002@sina.com (X.X.); yhl1209898426@126.com (H.Y.); zhanglq1999@163.com (L.Z.); 2 State Key Laboratory for Conservation and Utilization of Bio-Resources in Yunnan & School of Life Sciences, Yunnan University, Kunming 650091, China; W1bo1058@163.com (F.L.); yangding129@163.com (D.Y.); LCJ649954157LCJ@163.com (C.L.); 3 Lijiang Teachers College, Lijiang 674100, China; glcyhj@126.com

**Keywords:** Chinese cabbage, gene expression, male sterility, pollen development, plant hormone pathway

## Abstract

Male sterility exists widely in flowering plants and is used as a fascinating tool by breeders for creating hybrid varieties. Herein, stamen samples from male sterile CCR20000 and male fertile CCR20001 lines during two developmental stages were employed to elucidate the molecular changes during flower development in fertile and sterile Chinese cabbage lines. RNA-seq revealed weak transcriptional activity in the sterile line, which may have led to the abnormal stamen development. The differentially expressed genes were enriched in plant hormone, carbon metabolism, and biosynthesis of amino acid pathways. Important genes with opposite patterns of regulation between the two lines have been associated with the male sterility trait. Members of the transcription factor families such as AP2, MYB, bHLH, and WRKY were highly active in the regulation of structural genes involved in pollen fertility. This study generated important genomic information to support the exploitation of the male sterility trait in Chinese cabbage breeding programs.

## 1. Introduction

Male sterility exists widely in flowering plants and is used as a fascinating tool by breeders for creating hybrid varieties. The hybrids produced through male sterility have heterotic vigor, high uniformity, tolerance to environmental challenges and high yield [1]. Chinese cabbage (*Brassica rapa* ssp. *pekinensis*) is the most important species of the *Brassicaceae* family [2]. It is widely cultivated in most parts of China, especially in the Yangtze-Huaihe River Basin. It has become famous in many countries owing to the fact that it has a fascinating shape and is enriched with vitamins (A, C, B6 and K) and minerals such as iron and calcium [3]. Many cabbage cultivars are produced by crossing male sterile lines with pollination lines for heterosis [4]. Recently, the cytoplasmic male sterility (CMS) technique has been widely used in cabbage breeding to produce high yielding hybrids to meet the nutritional requirements of the growing population [5].

CMS was found to be successful in more than 150 plant species, including rice, soybean, pearl millet, ramie and wheat etc. [6,7,8,9], which have evolved through spontaneous mutations, induced mutations, wide hybridization and protoplast fusion [10]. CMS occurs due to changes in the mitochondrial genes rather than the nuclear genes [5]. CMS plants exhibit reproductive irregularities, i.e., abnormal anthers, non-functional pollen, and carpelloid or petaloid stamens. Approximately, there are 28 different CMS in 13 plant species due to the rearrangement of at least 10 mitochondrial genes [11].

Numerous pathways are involved in anther development. The plant hormone signaling pathway and different phytohormones synthesis were identified to be significantly altered in CMS plants [12]. Similary, higher Abscisic acid (ABA) and lower indole acetic acid (IAA) contents were identified in different Chinese cabbage CMS lines compared with maintainer lines, indicating that ABA and IAA have different roles in fertile pollen development [13]. A cluster of genes involved in different pathways related to the anther development, i.e., lipid transport and metabolism and carbohydrate metabolism, was also reported [14,15]. For example, in kenaf, the ATPase subunit 6 (*atp6*), which is a dynamic mitochondrial gene involved in the energy supply, was found to be responsible for male sterility [16], whereas the gene *CaMF2a* (lipid transfer protein) was revealed to control pollen development in *Capsicum annum* L. [17].

Additionally, transcription factors (TF) were found to be involved in pollen infertility. Pollen development has been broadly studied in model plants, i.e., Arabidopsis and rice [18]. TFs, *DYSFUNTIONAL TAPETUM1* (*DYT1*) and *DEFECTIVE TAPETUM DEVELOPMENT AND FUNCTION1* (*AtTDF1*), *AtMYB35*, *bHLH89*, *bHLH91*, *MYB80* and *MYB103* were identified to regulate pollen development or be involved in related mechanisms [19,20,21]. *AtDYT1* encodes a bHLH89/91 protein that interacts with the AtAMS protein and regulates *AtMYB80* expression [22]. Likewise, a similar signaling pathway including *UDT1*-*TDR1*-*TIP2*-*EAT1* transcriptional cascade is also involved in the regulation of pollen development in rice [22]. *OsUDT1* (*UNDEVELOPED TAPETUM1*) is a homolog of *AtDYT1*. This detailed description emphasizes that the signaling pathway regulating pollen development is highly conserved in plants [23].

In order to elucidate the mechanisms of male sterility and identify associated gene clusters in Chinese cabbage, it is important to implement a comparative study of male fertile and sterile cultivars. Previous studies in Chinese cabbage revealed that a cluster of TFs and cell wall responsive genes were upregulated in the male fertile line relative to the sterile line [24], whereas downregulation of sucrose and starch metabolism-responsive genes led to the reduced sucrose and sugar consumption, ultimately resulting in a decline in fertility [24,25].

In the current study, the objective was to reveal the DEGs and key pathways affecting the sterility trait in two specific Chinese cabbage lines through RNA-Seq. Based on computational analysis, candidate genes representing the different molecular mechanisms involved in male sterility and fertility were assessed. Our findings provide useful information for future endeavors focused on Chinese cabbage heterosis breeding.

## 2. Materials and Methods

### 2.1. Plant Material and Growth Conditions

**Figure 1:**
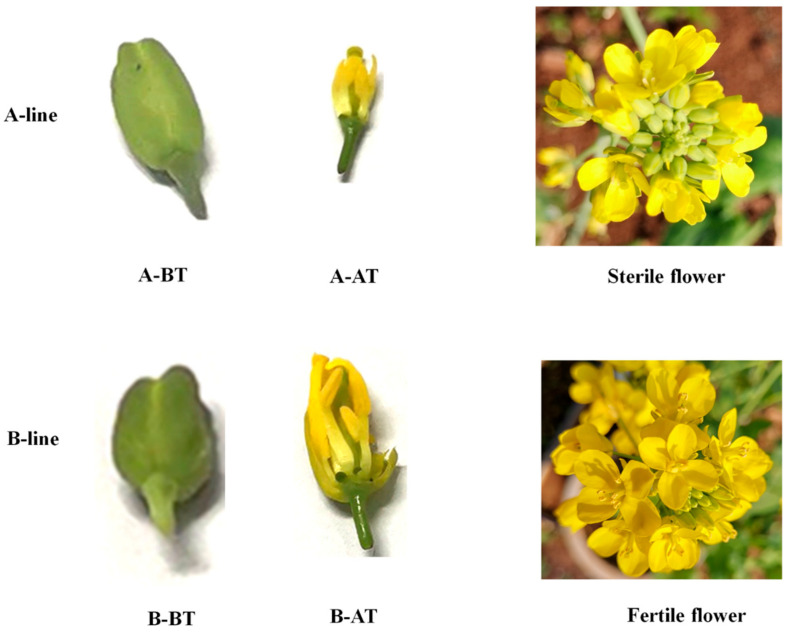
Morphological comparison of male sterile and male fertile Chinese cabbage lines before (BT) and after (AT) stamen maturation stages. The male sterile line (A-line) displays abnormal stamen development and sterile pollen. The male fertile line (B-line) shows proper stamen development and filament elongation and has fertile pollen.

The new Chinese cabbage male sterile line CCR20000 (A) and fertile line CCR20001 (B) developed by our group were employed in this study. Stamens samples were collcted as the experimental materials at two stages according to the developmental status of the stamens in the male sterile line (Figure 1). During the first stage (before stamen maturation, BT), a normal development of stamens could be observed, and the bud size was less than 1.5 mm in the A line. The second stage (after stamen maturation, AT) is characterized by abnormal stamen development with the size of the bud reaching 3 mm in the A line. In the fertile line (B), there was no abnormal development of the stamen. Both sterile and fertile lines harbor the same nuclear genome but different cytoplasm. The sterile floral petals were visually wrinkled and smaller than than the fetile flowers in the three replicates of male sterile lines. Stamen degeneration and shorter stamen length was observed in the sterile lines comparative to the fertile flowers. However, other characteristics are same in both sterile and fertile lines, e.g., bud opening and pistil [26]. The period of sampling was the same for both lines. In total, 12 samples (three biological replicates per line and per developmental stage) were collected, immediately frozen in liquid nitrogen and transferred to the refrigerator at −80 °C for preservation.

### 2.2. RNA Extraction and Illumina Sequencing

RNA extraction and transcriptome sequencing were performed as previously described [27,28]. Briefly, total RNA was isolated from the 12 samples through TRIZOL^®^ reagent (TIANGEN, Beijing, China) according to the manufacturer^’^s protocol. Extracted RNA from different samples was purified by using RNase-free DNase I (TaKaRa, Kyoto, Japan) to remove the genomic DNA contamination. Total RNA concentration in different samples was calculated using a NanoDrop microvolume spectrophotometer (Thermo Scientific NanoDrop Products, Waltham, MA, USA). Thereafter, the Illumina HiSeq4000 platform was used for RNA-Seq.

### 2.3. Analysis of the RNA-Seq Data

Quality reads of the raw RNA-Seq data were processed by the fastQC application v0.11.2 [29]. Low-quality reads and reads containing adapters were removed by the Trimmomatic (0.36.5) tool to reach the clean reads data [30]. Paired-end clean reads were aligned to the available reference genome https://www.ncbi.nlm.nih.gov/genome?LinkName=bioproject_genome&from_uid=472930 (accessed on 11 July 2020) using HISAT2 (2.1.0) [31]. StringTie (1.3.4) was employed to count the number of reads mapped onto each gene, and quantification of the gene expression level in the number of fragments per kilobase of the transcript sequence per million base pairs was sequenced (FPKM) [32]. Differential expression analysis was performed using the DESeq2 R package (2.11.38). Genes with *p*-value < 0.05 and −1 < log2 fold change < 1 were considered as significant differentially expressed genes (DEG) in comparative analysis.

### 2.4. Functional Classification and Pathway Enrichment Analysis

Functional enrichment analysis, including gene ontology (GO) analysis, was performed to identify which DEGs were significantly involved in each GO term. GO enrichment analysis was performed by AgriGO software [33]; GO term with FDR *≤* 0.05 was considered significantly enriched by DEGs. Additionally, the Kyoto encyclopedia of genes and genomes (KEGG) pathway analysis was executed to retrieve the enriched pathways.

### 2.5. RNA-Seq Data Evaluation

The expression pattern of ten randomly selected differentially expressed genes was assessed by qRT-PCR to validate the RNA-seq data. The primers of the nominated genes were designed using AmplifX 1.5.4. The *Actin* gene was used as an internal control in qRT-PCR; the reaction was performed in a 96-wells plate on an ABI prism 7500 Real-Time PCR system (Applied Biosystem, Foster City, CA, USA) using SYBR Green Master ROX (TaKaRa, Tokyo, Japan). The thermal conditions were 95 °C for 30 s, followed by 40 cycles of 95 °C for 10 s, 60 °C FOR 34 s and 72 °C for 15 s.

The relative expression level of the selected DEGs was calculated with the 2^−*ΔΔCT*^ method.

## 3. Results

### 3.1. Overview of the RNA-Seq and Differential Genes Expression Analyses

Two Chinese cabbage cultivars with male sterile flowers CCR20000 (A) and fertile flowers CCR20001 (B) were used in this study. We observed the male reproductive organs at two developmental stages between male sterile and fertile lines before (BT) and after (AT) stamen maturity (Figure 1). The male reproductive organs developed normally in the two lines at the BT stage. However, after stamen maturity (AT), an abnormal stamen development was observed in the A line with no mature pollen produced, whereas normal stamen development and fertile pollen were observed in B line. Compared with the maintainer line B, the sterile line A had slightly smaller flower buds, highly degenerated stamens, and short anthers (Figure 1).

**Figure 2:**
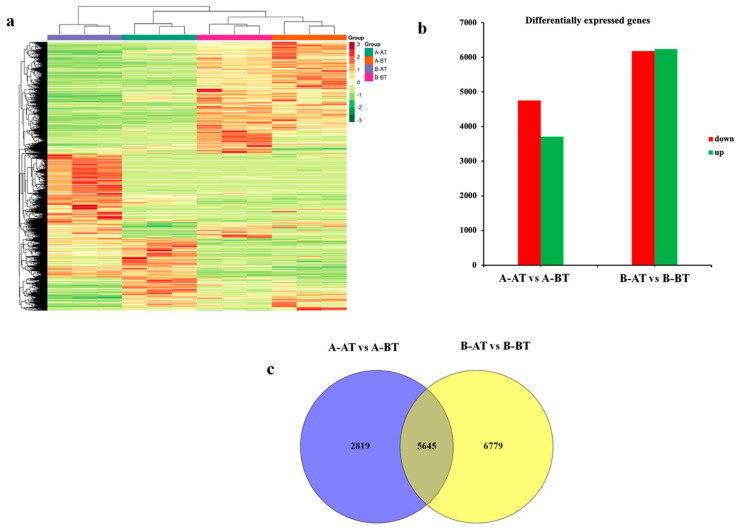
An overview of the comparative gene expression difference between male sterile and fertile lines in different comparisons. (**a**) Heatmap illustrating the FPKM based expression pattern of all significantly expressed genes before and after stamen maturation in male sterile and fertile lines. (**b**) Bar graph exhibiting the up and down regulation of the genes in the different comparisons. (**c**) Venn diagram representing the overlapping differentially expressed genes (DEGs) in the two representative comparisons.

RNA-seq analysis of A-BT, B-BT, A-AT and B-AT with three biological replicates was performed in this study. After filtering out the low-quality reads and adapters, the cleaned reads ranged from 40,714,802 to 46,215,568 and were mapped onto the reference genome (https://www.ncbi.nlm.nih.gov/genome?LinkName=bioproject_genome&from_uid=472930) (accessed on 11 July 2020) using HISAT2. The range of successfully aligned reads to the reference genome was from 92.92 to 92.30%, indicating very high-quality mapping (Supplementary Table S1). The gene expression was quantified as number of fragments per kilobase of the transcript sequence per million base pairs sequenced (FPKM), and a hierarchical clustering heatmap was constructed based on the FPKM profile of the different samples (Figure 2a). As shown in Figure 2a, all biological replicates were clustered together, indicating high-quality transcriptome sequencing data. Additionally, it could be observed that the B-AT samples (samples collected from the male fertile line after stamen maturity) were separated from the other samples, indicating that major molecular changes between sterile and fertile samples occurred at the stamen maturity stage.

The differentially expressed genes (DEGs) analyses between the two comparisons, e.g., A-BT_vs_A-AT and B-BT_vs_B-AT were investigated using DESeq2. Genes were considered as DEGs if they have a *p*-value < 0.05 and −1 < log2 fold change < 1. Among 8464 DEGs, 4758 genes were identified to be upregulated, and 3,706 were downregulated in A-AT relative to A-BT (Figure 2b, Supplementary Table S2), whereas 6182 genes were upregulated and 6242 genes were downregulated in B-AT compared with B-BT. The lower number of DEGs observed in line A as compared with line B suggests weak transcriptional activity, which may have led to the abnormal stamen development, resulting in male sterility. A comparison of the two lists of DEGs from A and B showed that there were 5645 common DEGs, which may play key roles in stamen development in Chinese cabbage (Figure 2c). In addition, we identified 6779 specific DEGs in the fertile line B, representing potential genes sustaining the normal growth of stamens.

**Figure 3:**
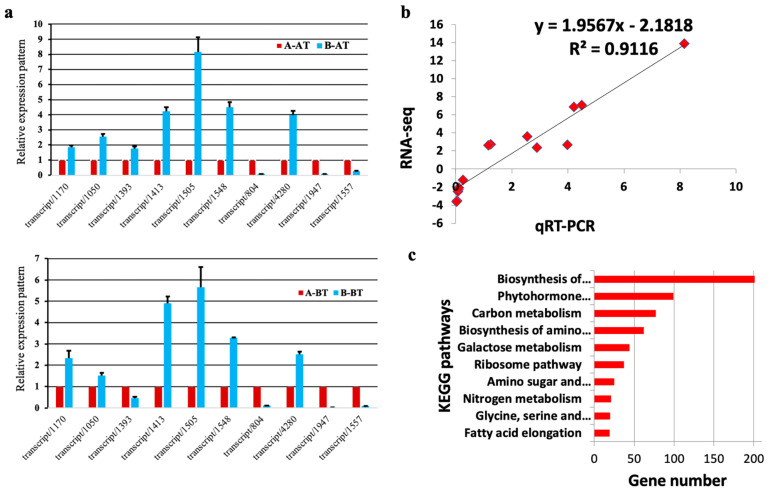
Validation of gene expression profile and KEGG enrichment analaysis. (**a**) qRT-PCR expression pattern of nominated genes—the error bars represent the standard deviation from three technical replicates and three biological replicates; (**b**) correlation plot between qRT-PCR and RNA-seq expression profiles based on ten selected DEGs; (**c**) Top 10 enriched KEGG pathways.

We randomly selected ten DEGs for qRT-PCR to evaluate the validity of the RNA-Seq data. The qRT-PCR expression patterns of the ten DEGs in male sterile and fertile lines at BT and AT stages followed the same expression pattern as per RNA-Seq data (R^2^ = 0.91) (Figure 3a,b, Supplementary Table S3). This result confirms that our RNA-seq expression data and subsequent interpretations are reliable.

We further performed a KEGG enrichment analysis of the DEGs. The top ten most enriched pathways were presented in Figure 3c. Apart from biosynthesis of the secondary metabolites pathway, phytohormone biosynthesis, carbon metabolism, biosynthesis of amino acids, galactose metabolism, amino sugar and nucleotide sugar metabolism pathways were significantly enriched and are predicted to play key roles in pollen fertility in Chinese cabbage.

### 3.2. Differentially Expressed Transcription Factors

Transcription factors (TFs) play key roles in plant development and gene expression regulation [34,35]. We retrieved the TFs through BLASTX and cross-checked to the plant transcription factor database. We identified 596 TFs distributed into different families (Supplementary Table S4) with the most represented ones being apetala 2 (AP2, 87 transcripts), myeloblastosis and myeloblastosis-related (MYB and MYB-related, 80 transcripts), NAC (47 transcripts), MADS (38 transcripts), basic helix-loop-helix (bHLH, 37 transcripts), and WRKY (29 transcripts). B-AT samples had a high expression pattern for several members of AP2, NAC, and MYB compared with A-AT, indicating that these TFs represent active members of pollen fertility in Chinese cabbage. We speculate that these TFs may be involved in the regulation of structural genes associated with pollen development and fertility in Chinese cabbage.

### 3.3. Genes Related to Phytohormones

**Figure 4:**
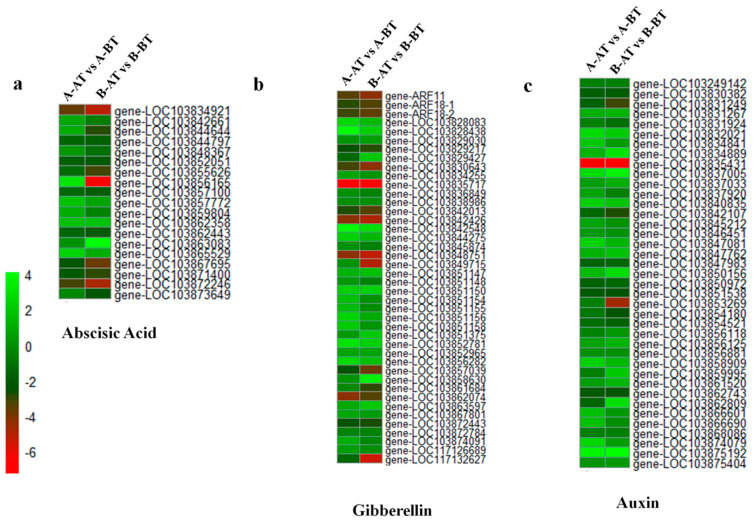
Heatmap exhibiting the expression pattern of plant hormone-responsive genes: (**a**) abscisic acid, (**b**) gibberellins, and (**c**) auxin in Chinese cabbage male sterile and fertile lines. The up and down regulations of DEGs are represented by green and red color bars, respectively.

Plant hormones play an important role in plant growth and development and are known be involved in metabolic pathways associated with pollen fertility [36]. Significant abscisic acid (ABA) accumulation in anthers leads to pollen abortion in plants [37]. There were 19 ABA-responsive DEGs retrieved in this study, with various expression patterns in the different samples (Figure 4a, Supplementary Table S5). Globally, ABA-responsive genes were up-regulated from the stage BT to AT in both Chinese cabbage lines. However, most of the DEGs were higher expressed in A samples than in B samples, which may indicate a higher accumulation of ABA in A samples after stamen maturation compared with B samples. Some specific genes, such as *gene-LOC103842661*, *gene-LOC103844644*, *gene-LOC103848367*, *gene-LOC103856165* and *gene-LOC103859804*, displayed opposite patterns of regulation between A and B lines and are predicted to play major roles in ABA-induced regulation of pollen fertility in Chinese cabbage. Apart from ABA, 42 auxin responsive DEGs were identified in A and B Chinese cabbage lines at the two developmental stages (Figure 4b, Supplementary Table S5). Among the 42 auxin DEGs, 10 genes were downregulated, and 32 genes were upregulated in the fertile B line after the stamen maturity stage (AT) relative to the sterile line A at AT stage. Since the auxin hormone contributes to the development and elongation of stamina and promotes maturation of anthers and pollen [38], we deduce that the strong downregulation of auxin-related genes in the sterile line could be an important factor of the male sterility trait. Additionally, 39 gibberellin acid (GA)-responsive DEGs were also retrieved, with most of these genes being higher induced in B-AT samples relative to A-AT samples (Figure 4c, Supplementary Table S5). Two DEGs (*gene-LOC103853269* and *gene-LOC103862809*) showed opposite regulation patterns between the two lines and could be important genes for future functional studies. Collectively, we conclude that variations in phytohormones affect pollen fertility in the stamen of Chinese cabbage.

### 3.4. DEGs Involved in Carbon Metabolism

**Figure 5:**
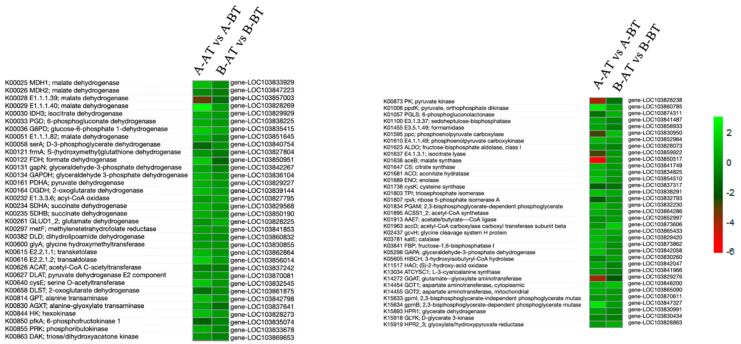
Heatmaps illustrating expression patterns of Carbon metabolism pathway-responsive DEGs in male sterile and fertile lines of Chinese cabbage. The annotation of the each gene playing a role in the carbon metabolism is described in detail. The side color ribbon represents the upregulation and downregulation of DEGs.

Pollen fertility and microspore development are regulated through an abundance of starch accumulation during microsporogenesis. Several genes related to carbon metabolism exhibited differential expression in male sterile and fertile lines at the tw developmental stages (Supplementary Table S6). The genes participating in carbon metabolism are involved in sucrose metabolism, pentose and glucuronate inter-conversions, and amino sugar and nucleotide sugar metabolism [39]. There were 69 DEGs identified in this study, and they participate in different compartments of the carbon metabolism, such as formaldehyde, cysteine, succinate and Glucose-6P (Figure 5). Importantly, we observed that many DEGs showed opposite patterns of regulation between the two lines. Particularly, the downregulated genes in the B line may negatively modulate pollen development and filament elongation in Chinese cabbage.

### 3.5. DEGs Related to the Biosynthesis of Amino Acids 

**Figure 6:**
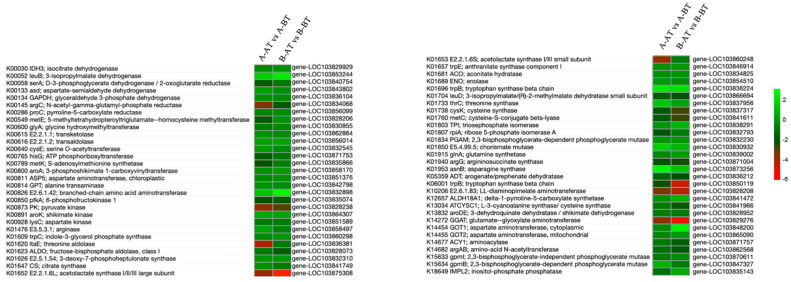
Heatmaps exhibiting DEGs’ enrichment in the biosynthesis of the amino acids pathway. Heatmaps show the expression patterns of DEGs in the given Chinese cabbage lines.

Amino acids are synthesized from precursor molecules via different steps, as shown in the Figure 6. Precursor molecules such as pyruvate, oxaloacetate, oxoglutarate play important functions in developing the anthers of fertile plants [40]. There were 59 DEGs involved in the amino acid biosynthesis pathway (Figure 6, Supplementary Table S7). Among them, 24 genes were upregulated and 25 genes were downregulated in A-AT vs A-BT. Thirty genes were upregulated while 19 genes were downregulated in B-AT vs B-BT. Four genes, including *gene-LOC103853244* (Isopropylmalate dehydrogenase), *gene-LOC103832898* (aminotransferase), *gene-LOC103836224* (tryptophan synthase beta chain), and *gene-LOC103848200* (asparate aminotransferase), were found to be higher expressed in the B-AT sample than the A-AT sample and may be important for pollen development and fertility in Chinese cabbage.

### 3.6. Genes Related to Anther and Pollen Development

In this study, all of the DEGs were annotated against the processes of anther and pollen development in *Arabidopsis thaliana*, resulting in the identification of 78 DEGs predicted to have various functions in pollen and anther development (Supplementary Table S8). Most of the DEGs were upregulated in the fertile line samples compared with the sterile line samples. In addition, out of the 78 DEGs, 22 genes displayed a differential regulation pattern with a conspicuous discrepancy between the two lines. Further functional characterizations of these 22 genes will clarify their potential roles for pollen fertility in Chinese cabbage.

## 4. Discussion

In higher plants, male sterility is a common phenotypic trait in which the abortion of stamens occurs. This affects the plant’s ability to produce fertile anthers, pollen or make gametes under typical natural conditions [41,42]. As a male gamete plays a key role in plant inheritance, utilization of male sterile lines in Chinese cabbage is a better way to produce hybrid seeds compared with the traditional breeding system. RNA-seq has been employed to determine the putative DEGs that regulate the sterility and fertility in different plant species [43,44]. In this study, we observed that male sterility mainly occurs after the stamen maturation stage in the new Chinese cabbage line CCR20000. We identified several DEGs involved in plant hormone transduction, carbon metabolism, the biosynthesis of amino acids, and anther and pollen development.

In plants, flower development is strongly influenced by hormonal regulation [45]. Previous studies indicate that the genes involved in hormone signaling play important roles in plant sex determination [46]. Auxin responsive hormones provoke a critical regulatory function in the process of floral growth and development in plants [38]. Several pollen mutant analyses revealed that auxin is also required for a number of later events during stamen development. In our study, several auxin responsive genes, such as *gene-LOC103858630* (SAUR36) and *gene-LOC103829427* (SAUR61), were upregulated in the fertile line compared with the sterile line, indicating that these genes have great importance in pollen maturation. Gibberellic acid (GA) is a growth regulator and participates in anther and pollen development. GA is vital to anther development as well as pollen viability [47]. The deficiency in GA results in male sterility or shortens the time of anthesis in cucumber, hemp and spinach [48], whereas male fertile flowers are promoted by exogenous application of GAs in cucumber [49]. Similar to auxin-related DEGs, we also identified several GA-related DEGs with many genes higher expressed in B-AT samples compared with A-AT samples. These results show that a weak GA accumulation in Chinese cabbage samples may result in pollen sterility.

Abscisic acid (ABA) is involved in various aspects of anther and pollen development, including the programmed cell death of tapetum and maturation of the anther wall [50]. High accumulation of ABA is detrimental to pollen viability and fertility [37]. In this study, we observed that most ABA-responsive DEGs, including abscisic acid receptor PYL9 and ABSCISIC ACID-INSENSITIVE 5-like protein 2, were higher expressed in sterile line samples compared with fertile line samples. This will probably lead to high ABA levels in the sterile samples and affect pollen fertility [51]. Overall, given the evidence of plant hormones in the restoration of pollen fertility and male gamete development, we can conclude that plant hormones play an important role in male fertility in Chinese cabbage. Further quantification of the phytohormone levels in these lines at the two developmental stages will validate the results obtained from the RNA-seq study.

Transcription regulation is a fundamental process in all living organisms [52]. Transcription factors (TFs) can regulate multiple downstream genes, essential components of cellular machinery, and play key roles in plant growth and development [53]. In the current study, different TFs belonging to diverse families were identified as DEGs, and the dominant families were AP2, MYB, NAC, MADS, bHLH, WRKY. Previous studies also reported that the AP2 gene family is highly active in pollen fertility in various plants. However their specific gene functions and regulatory networks are yet to be investigated [54,55]. The bHLH proteins bind to the specific DNA target sites and play a critical role in programmed cell death (PCD) and pollen development [56]. The bHLH-MYB complex activates the expression of downstream genes essential for fertile stamen development in Arabidopsis [56]. In maize, *bHLH27* regulates the chalcone synthase gene important to the flavonoid metabolism and with key role in pollen development and filament elongation [57]. Moreover, three genes, *bHLH10*, *bHLH60* and *bHLH90*, were positively coexpressed in Arabidopsis anther, but the mutant of each gene led to the defective anther phenotype relative to the fertile anther, indicating that bHLHs redundantly participate in male fertility [58]. In this study, these bHLH genes were also upregulated from the stage BT to AT in both lines, but higher expression levels were observed in the fertile line B. *MYB21* and *MYB24* activate the expression of various genes essential for jasmonic acid regulation, playing role in anther development and filament elongation in Arabidopsis [59]. Likewise, *AtMYB4* was found to be strongly induced in the fertile stamens comparative to the sterile stamens in Arabidopsis [60]. *GhMYB24* in cotton is mainly localized in the cell nucleus and is found to have strong expression in anther and viable pollen grains [61]. In concordance with these reports, in our experiment, *MYB4* (*gene-LOC103844065*) and *MYB21* (*gene-LOC103837005*) were found to be upregulated in the fertile samples at stamen maturity (B-AT) compared with the A-AT samples. These genes represent important molecular tools for manipulating the male sterility trait in Chinese cabbage. WRKY TFs are involved in a wide range of biological processes including pollen development [62]. It has been shown that *WRKY6*, *WRKY42* and *WRKY46* were downregulated in the fertile stamens of *Brassica compestris* [63]. Similarly, we observed a significant downregulation of *WRKY6* and *WRKY42* in samples of the fertile B line compared with the sterile A line, indicating that these genes might be linked to the male sterility trait in Chinese cabbage. There are currently several RNA-seq data available on Chinese cabbage’s sterile and fertile lines from different research groups and at various growth stages. We propose that these datasets could be exploited for a large-scale gene co-expression network project in order to delineate the important gene modules associated with the male sterility trait and the master regulators [64].

In the carbon metabolism pathway, pyruvate kinase is an enzyme catalyzing the conversion of phospoenolpyruvate and ADP to pyruvate and ATP in glycolysis, which have role in regulating cell metabolism [65]. Pyruvate kinase responsive gene, *gene-LOC103828238*, was downregulated in the male sterile line, which may have affected ATP formation and physiological processes in mitochondria. The observed downregulation of pyruvate kinase genes might have led to decreased carbohydrate accumulation in the flowers of the cabbage male sterile line, as per a previous study where the male sterile line in broccoli had weak carbohydrate accumulation [41]. The gene, i.e., *gene-LOC103864307*, encoding shikimate kinase was identified to be upregulated in fertile B-AT and downregulated in A-AT. As per the literature, shikimate kinase catalyzes the shikimate pathway, which is involved in the advanced developmental stages of pollen production and receptive stigmas [66]. Moreover, citrate synthase (*gene-LOC103841749*) and cysteine synthase (*gene-LOC103837317*) were found to be more induced in the male fertile line than in the male sterile line. It has been reported that citrate synthase is the initial enzyme of tricarboxylic acid, an energy source for pollen development [67]. Additionally, in accordance with our results, cysteine synthase was observed to be highly expressed in the fertile line compared with the male sterile line of pepper [68].

## 5. Conclusions

A comparative transcriptome study was performed using Chinese cabbage male sterile and fertile lines before and after stamen maturation. Through comparative analysis, differentially expressed genes, including transcription factors, plant hormones and other cluster of genes involved in the biosynthesis of amino acid and carbon metabolism, were identified, and their roles in male fertility development were predicted. Our findings provide a foundation for the functional characterization of the key genes involved in fertile pollen development and related mechanisms in Chinese cabbage. It is expected that our results will improve the efficiency of hybrid seed production in Chinese cabbage breeding programmes.

## Data Availability

All the raw reads are submitted to the NCBI SRA with the accession number PRJNA671749.

## Supplementary Materials

The following are available online at https://www.mdpi.com/article/10.3390/life11060525/s1, Supplementary Table S1. Summary of RNA-Seq data; Supplementary Table S2. An overview of significant DEGs present in male sterile and male fertile lines. All the significant genes are screened based on the *p* value < 0..5 and annotations of each gene are given in the table; Supplementary Table S3. Primer sequences of the genes used for qRT-PCR; Supplementary Table S4. Description of differentially expressed TFs in male sterile and fertile lines before and after the stamen maturity; Supplementary Table S5. Description of significant phytohormone responsive genes in male sterile and fertile lines of Chinese cabbage; Supplementary Table S6. An overview of DEGs involved in carbon metabolism pathway; Supplementary Table S7. An overview of the biosynthesis of amino acids pathway exhibiting the genes expression difference in comparative analysis of male and female cabbage lines; Supplementary Table S8. Detailed list of DEGs involved in pollen development and fertility in Chinese cabbage lines.
